# Pleural Effusion After Endoscopic-Assisted, Minimally Invasive Off-Pump Bypass Grafting

**DOI:** 10.3390/jcdd13070326

**Published:** 2026-07-13

**Authors:** Fleur Sampon, Antonius Johannes van de Poll, De Qing Görtzen, Joost F. J. ter Woorst, Pim A. L. Tonino, Ferdi Akca

**Affiliations:** 1 Department of Cardiothoracic Surgery, Catharina Hospital, Michelangelolaan 2, 5623 EJ Eindhoven, The Netherlandsdeqing.gortzen@catharinaziekenhuis.nl (D.Q.G.); joost.t.woorst@catharinaziekenhuis.nl (J.F.J.t.W.); ferdi.akca@catharinaziekenhuis.nl (F.A.); 2 Department of Cardiology, Catharina Hospital, 5623 EJ Eindhoven, The Netherlands; pim.tonino@catharinaziekenhuis.nl; 3 Department of Biomechanical Engineering, Eindhoven University of Technology, 5631 BN Eindhoven, The Netherlands

**Keywords:** minimally invasive, pleural effusion, coronary surgery

## Abstract

Postoperative pleural effusion (PE) is a common finding after endoscopic-assisted, minimally invasive coronary bypass surgery (Endo-CAB). This study evaluated its postoperative course, defined a threshold for clinically significant PE, and identified associated risk factors. Between May 2021 and January 2026, 727 patients were retrospectively analyzed. A total of 3141 postoperative chest X-rays obtained during hospitalization and follow-up were reviewed to quantify PE. Clinically significant PE was defined as ≥25% on radiographic assessment. Baseline characteristics and postoperative outcomes were analyzed, and logistic regression was used to identify independent risk factors. All patients received oral diuretics for two weeks after discharge as standard protocol. PE occurred in 86.1% of patients, typically developing within the first two postoperative days, with a median maximum of 13% [5–21%]. By the third postoperative week, PE had resolved to 0% [0–0%] in most cases. Adjustment of diuretic therapy was required in 18.3%, while 5.1% underwent thoracocentesis. Clinically significant PE occurred in 15.4% and was associated with longer hospital stay and increased blood transfusion rates. Independent risk factors included age, body mass index, continuation of dual antiplatelet therapy, and blood transfusion. Overall, PE is frequent but usually self-limiting after Endo-CAB.

## 1. Introduction

Minimally invasive coronary bypass surgery is increasingly performed and shows comparable long-term outcomes to traditional median sternotomy, while improving short-term outcomes [1,2,3,4,5]. With these new approaches, there is also attention to further optimize postoperative care [2,3,4,5,6,7,8]. Notably, a higher incidence of unilateral left-sided pleural effusion (PE) has been observed, which may contribute to prolonged hospitalization and necessitate invasive interventions such as thoracocentesis [7,8]. Previous research has shown a higher incidence of PE after minimally invasive techniques compared to median sternotomy [3,9,10].

This study aims to explore the course of postoperative PE after endoscopic-assisted coronary bypass surgery (Endo-CAB), a minimally invasive procedure that utilizes endoscopic mammary artery harvesting with off-pump bypass grafting via a small thoracotomy (4–5 cm), using routine thoracoscopic instruments [2]. Secondly, it aims to define a cut-off value for clinically significant PE and to identify risk factors for the development of clinically significant PE during follow-up.

## 2. Study Design and Population

### 2.1. Materials and Methods

From May 2021 until January 2026, patients who underwent an Endo-CAB procedure at the Catharina Hospital (Eindhoven, The Netherlands) were retrospectively analyzed. Patients who underwent conversions to a median sternotomy, previous heart surgery or a preoperative elevated hemidiaphragm were excluded. Patients who underwent either single-vessel(LIMA to LAD), multivessel bypass grafting, hybrid revascularization (Endo-CAB LIMA to LAD combined with percutaneous coronary intervention (PCI)) and concomitant arrhythmia surgery were included in this study. All patients were evaluated by a heart team, which included a cardiac surgeon and an interventional cardiologist, to assess the need for surgical revascularization. Patients with single-vessel disease of the LAD, for whom either PCI or Endo-CAB was technically feasible, were eligible for PCI or Endo-CAB. Our institution’s protocol is that patients with double-vessel disease are planned for minimally invasive surgery (if the urgency permits). Hybrid revascularization was chosen in high-risk patients or with a non-LAD target less suitable for a bypass graft with a focal stenosis, well treatable with PCI.

Per institutional protocol, acetylsalicylic acid (ASA) therapy was continued perioperatively in all patients. Patients who had undergone PCI within three months before surgery remained on dual antiplatelet therapy (DAPT). In all other patients, DAPT was withheld perioperatively, with ticagrelor discontinued three days and clopidogrel five days prior to surgery. Direct oral anticoagulants (DOACs) and vitamin K antagonists were discontinued before surgery and resumed at least three days postoperatively.

**Figure 1:**
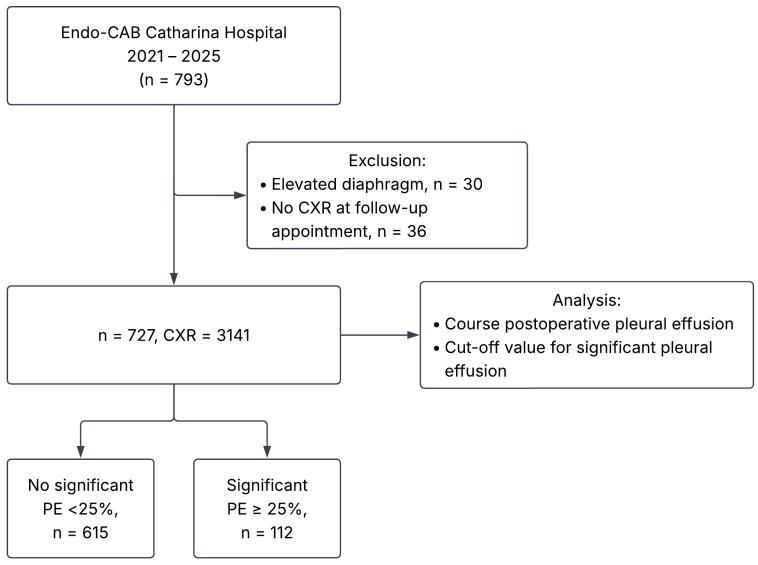
Flowchart of patient and chest X-ray selection. CXR = chest X-ray; PE = pleural effusion.

Patient selection is displayed in Figure 1. Patients were routinely discharged 3–4 days after surgery [2]. Furthermore, at our institution we implemented a very early drain removal protocol following Endo-CAB, designed to promote Enhanced Recovery After Surgery (ERAS). Implantation of this protocol has been published by our group and is not associated with an increase in PE [11].

The drain is removed one hour after extubation if the production is less than 50 mL/hour without air leakage. If there is more than 50 mL/hour production, the drain remains in place until the following morning. As part of our protocol, all patients receive furosemide once daily 40 mg if no PE was present on the chest X-ray (CXR) on postoperative day (POD) 3. If any PE is detected, the furosemide dosage is increased to twice daily for 14 days.

**Figure 2:**
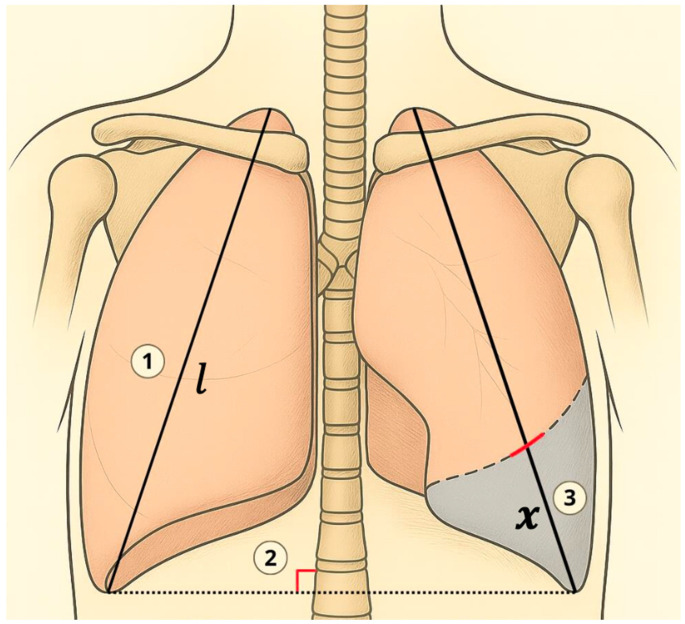
Method used to quantify pleural effusion. The dotted line signifies the line drawn from the deepest point of the right pleural sinus perpendicular to the spine to project the (supposed) position of the left-sided pleural sinus. From here, a line was drawn towards the left apex, ending at the lower limit of the left lung denoted by the dashed line, which was used as a measurement of the amount of pleural effusion (x). The red line is the spot where both lines cross.

Postoperative CXRs were routinely made on POD 0 and 3 and at our outpatient clinic. Additional CXRs were made based on clinical indication. In this study, patients were analyzed until 90-day follow-up or if the following events occurred: therapeutic thoracocentesis or operative intervention. CXRs were analyzed for the quantity of PE using the following method (Figure 2): in case of a clear left-sided pleural sinus, the amount of pleural effusion was classified as 0%. In case of left-sided pleural effusion (defined as the absence of a clear pleural sinus), the length of the right lung was determined by measuring the distance from the deepest point of the right pleural sinus to the apex of the right lung (l). From the deepest point of the right pleural sinus, a line perpendicular to the spine was drawn to project the (supposed) position of the left-sided pleural sinus. From here, a line was drawn towards the left apex, ending at the lower limit of the left lung, which was used as a measurement of the amount of pleural effusion (x). The percentage of volume loss caused by pleural effusion compared to the right lung was calculated using the following formula (Figure 2):

Percentage PE = x/l × 100%

One researcher reviewed all postoperative CXRs. In case of lack of clarity, a second researcher was consulted until a consensus was reached. If the pleural sinus or the top of the lung was not visible on the CXR, only this CXR was excluded.

### 2.2. Endpoints

The primary endpoint of this study was to objectify the course of postoperative PE and to describe the incidence of PE related interventions following Endo-CAB. Such as continuation or increase in diuretics and thoracocentesis. Drainage of PE is only performed in symptomatic patients or significant pleural effusion not responding to our standard medical therapy with diuretics.

Secondary outcomes were to identify a cut-off value for clinically relevant PE and to identify risk factors for the development of clinically relevant PE.

### 2.3. Statistical Analysis

A cut-off value for clinically significant PE was determined by plotting the cumulative number of interventions, stratified by changes in treatment strategy, against the percentage of PE observed at the first follow-up appointment.

Normality of the data was determined by the Kolmogorov–Smirnov test. Normally distributed continuous data was expressed as mean and standard deviation (SD), and an independent *t*-test was used to test for statistically significant differences. Non-normally distributed continuous data was expressed as median and interquartile range (IQR), and a Mann–Whitney U test was used for data comparison. Categorical data were expressed as frequencies and percentages, and were evaluated for statistically significant differences using Chi-square or Fisher’s exact test when appropriate. Furthermore, baseline characteristics and perioperative parameters underwent univariable and multivariable logistic regression to test for risk factors for clinically significant pleural effusion. In line with an exploratory model-building approach, a significance threshold of *p* < 0.100 was applied for variable selection to avoid prematurely excluding potentially relevant confounders or predictors. Data analysis was performed using SPSS Statistics for Windows, version 29.0.0.0 (241). Statistical significance was defined as *p* < 0.05 (two-tailed).

## 3. Results

Of the 793 patients during this study period, 30 patients (3.7%) had a postoperative elevated hemidiaphragm and 36 patients had no control CXR. Therefore, 727 patients were included in the analyses, with a total of 3141 CXRs assessed for the presence of pleural effusion. The mean number of CXRs performed per patient was 4.3. Postoperative PE was observed in 86.1% of the patients and 5.1% needed thoracocentesis.

### 3.1. Postoperative Course of PE

**Figure 3:**
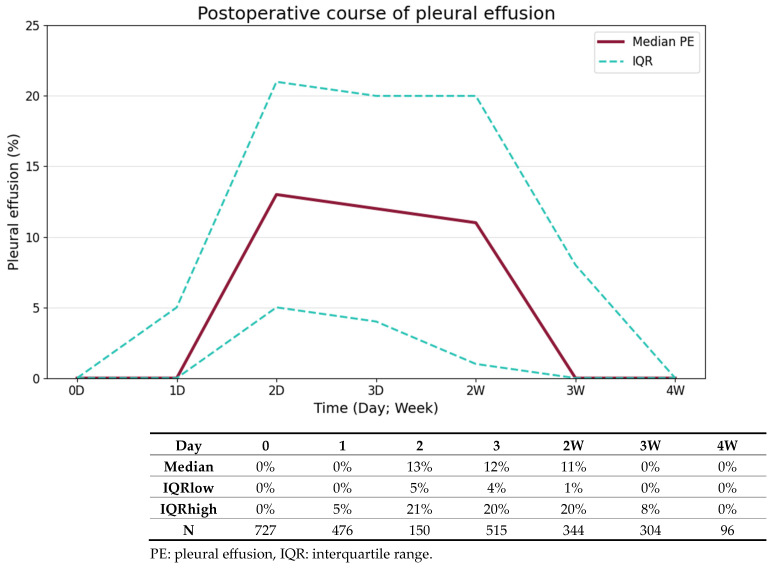
Postoperative course of pleural effusion after Endo-CAB surgery.

Median and IQR were calculated for POD 0, 1, 2, and 3, as well as for the follow-up appointments at 2, 3, or 4 weeks. As shown in Figure 3, no PE was observed on POD 0 (0% [0–0%]) and POD 1 (0% [0–5%]). With an increase at POD 2, the median PE was 13% [5–21%], which decreased to 0% [0–0%] at the follow-up appointments at 4 weeks.

### 3.2. Clinically Significant PE

**Figure 4:**
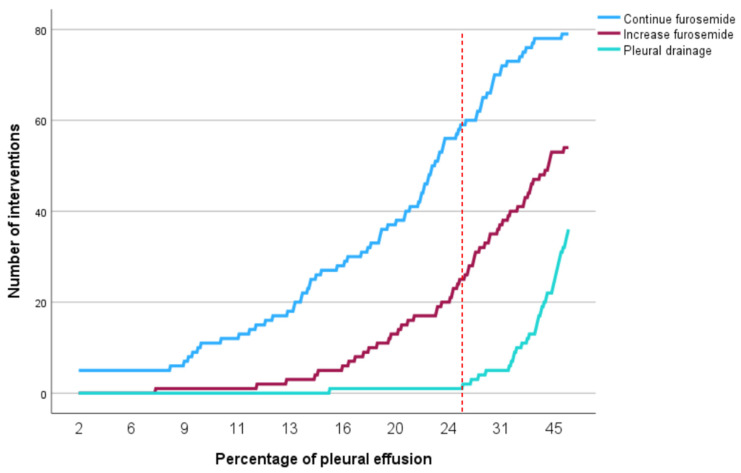
Cumulative changes in treatment plan at the first control appointment. Reference line for clinically significant pleural effusion at 25%.

A threshold of 25% was identified as clinically significant. This cut-off was chosen because the number of thoracocenteses increases substantially beyond this point, whereas PE below 25% can generally be managed with diuretic therapy alone (Figure 4).

The research population was divided into two groups: one group with clinically significant PE ≥ 25%, *n* = 112 (15.4%), and one group without clinically significant PE < 25%, *n* = 615 (84.6%). Patients with ≥25% PE were older (67 ± 8 vs. 65 ± 9 years, *p* = 0.028) and were more likely to have continued the use of dual DAPT (17.9% vs. 8.3%, *p* = 0.002). (Table 1) Furthermore, when comparing perioperative parameters, the group with ≥25% PE had a one-day longer hospitalization compared to the group with <25% PE (4 [3–5] vs. 3 [3–4] days, *p* < 0.001) and had a higher rate of blood transfusion (12.5% vs. 3.6%, *p* < 0.001). Notably, graft revision was required in four patients (0.7%) in the <25% PE group (Table 2). All four patients developed postoperative ischemia in the intensive care unit requiring graft revision. No sternotomy conversion was needed, and all revisions were performed through the Endo-CAB incision. In two cases, the original grafts were revised; in two others, a venous graft from the ascending aorta to the LAD was placed. The total dosage of furosemide given postoperatively did not differ between the groups. Change in treatment plan at the control appointment was higher for the group with ≥25% PE (81.3% vs. 12.7%, *p* < 0.001), with continuation of diuretics (21.4% vs. 8.9%), increasing the dosage of diuretics (28.6% vs. 3.6%) and the number of thoracocentesis (31.3% vs. 0.0%) (Table 3).

### 3.3. Logistics Regression Analysis

The results of multivariate logistic regression for significant PE are presented in Table 4. Age (1.03 [1.00–1.05], *p* = 0.021), BMI (1.06 [1.01–1.12], *p* = 0.027), continuation of DAPT (2.22 [1.19–3.91], *p* = 0.012) and blood transfusion (3.42 [1.65–7.08], *p* = 0.001) were found to be independent risk factors for the occurrence of significant PE (Table 4).

## 4. Discussion

This study represents one of the first evaluations of postoperative PE in modern minimally invasive coronary revascularization strategies. The primary objective of this study was to describe the course of postoperative PE after Endo-CAB, the secondary objective was to establish a cut-off value for clinically relevant PE and the identification of risk factors. In the majority of patients, postoperative PE normalized during the first follow-up appointment with the use of oral diuretics. For the whole population the rate of thoracocentesis after Endo-CAB was 5.1%. Furthermore, we found that PE < 25% can be effectively managed conservatively with a continuation/increase in diuretics and has a low risk of thoracocentesis during follow-up (*n* = 2, 0.3%). Finally, we found that age, continuation of DAPT, BMI and blood transfusion were independently associated with the incidence of significant PE. These factors can be used to develop better treatment plans and start early treatment of PE.

### Incidence and Severity of PE

The overall incidence of PE found in this study was 86.1%, which includes any amount of PE. Previous research regarding the incidence of PE after minimally invasive cardiac surgery is limited and does not use the same method to quantify PE. Studies regarding the incidence of PE after non-minimally invasive cardiac surgery, which uses blunting of the costophrenic angle as a marker for PE, report incidences ranging from 28 to 96% [9,12,13,14,15,16,17]. One explanation might be related to the minimally invasive nature of the Endo-CAB technique with a thoracotomy approach. This leads to surgical manipulation within the left pleural space and a reactive increase in postoperative pleural effusion. Furthermore, this technique involves routine CO_2_ insufflation during mammary artery harvest (capnothorax), which may contribute to pericardial and pleural inflammation. In Endo-CAB procedures, CO_2_ insufflation is applied under relatively higher intrathoracic pressure than other minimally invasive techniques such as mitral valve surgery, as the chest is fully closed during mammary artery harvest to optimize exposure and to maintain the operative field. Although its role in the development of PE remains speculative, this mechanism warrants further consideration. No recent research has been conducted regarding these topics, and older research showed conflicting results [13,15,17,18,19,20].

The most important endpoint for significant PE is the need for drainage. Our study found that 5.1% of patients required a thoracocentesis, which is comparable to the 4.2–14.4% found in previous studies [9,18,19,20]. Despite the high incidence of PE, frequent invasive intervention is not required and can be managed with diuretics in the majority of patients.

The literature regarding postoperative PE after minimally invasive coronary surgery remains scarce, and most available evidence originates from conventional CABG populations. Several mechanisms have been proposed for postoperative PE, including pleural and pericardial inflammation, surgical pleurotomy, postoperative bleeding, fluid shifts, and impaired lymphatic drainage. There was no significant difference in PE incidence between patients undergoing CABG with saphenous vein grafting alone versus additional IMA grafting, suggesting that postoperative inflammatory processes may play an important role independent of conduit choice [14,15,21,22]. In contrast, minimally invasive approaches such as Endo-CAB may introduce additional factors, including thoracotomy-related pleural manipulation and capnothorax during mammary artery harvesting. However, evidence directly comparing PE mechanisms anad incidence between minimally invasive and sternotomy approaches remains limited and heterogeneous.

The exploratory 25% threshold used in this study was selected as a pragmatic, cohort-derived cut-off associated with a clear change in postoperative management, including escalation of diuretic therapy and thoracocentesis. However, alternative definitions of clinically significant PE may be considered, such as defining significance based solely on the need for thoracocentesis. While thoracocentesis represents a clinically meaningful endpoint, our approach aimed to identify earlier clinically relevant PE that may benefit from closer monitoring or proactive treatment before invasive intervention becomes necessary.

## 5. Conclusions

This study demonstrates that PE is a frequent finding after Endo-CAB surgery; however, a minority of patients require invasive treatment. In this cohort, pleural effusions below the exploratory 25% threshold were generally managed successfully with diuretic therapy alone. Age and the continuation of DAPT were found to be independent risk factors for the occurrence of clinically significant PE (≥25%). These findings may help optimize postoperative care and timely intervention in patients undergoing minimally invasive coronary artery bypass grafting.

## 6. Limitations

There is no gold standard for quantifying the percentage of PE on a CXR; validated measurement methods are available only for thoracic CT scans. While CT-based assessment would be suitable for a prospective study, it was not feasible in this retrospective analysis. Therefore, we developed our described standardized technique for measuring PE. However, this method has inherent limitations, as postoperative anatomical changes after LIMA harvesting, including subtle or transient elevation of the left hemidiaphragm, may complicate the distinction between diaphragm contour and PE on plain chest radiography. Although patients with evident hemidiaphragm elevation were excluded, minor elevation may still have affected measurements in selected cases. In addition, the measurement technique was not formally validated against CT- or ultrasound-based quantification methods, and interobserver variability was not systematically assessed. Consequently, the reproducibility and accuracy of the proposed method remain uncertain and should be evaluated in future prospective studies. Future validation against CT- or ultrasound-based quantification methods would strengthen the accuracy and reproducibility of this approach.

As all patients received postoperative furosemide according to institutional protocol, the observed course of PE represents a treatment-modified rather than spontaneous natural course; this should be considered a potential confounder.

Furthermore, as this study reflects the experience of a single high-volume minimally invasive center with center-specific postoperative management protocols, including early drain removal and routine postoperative diuretic therapy, the generalizability of these findings to other institutions may be limited. Implementation of such protocols should be accompanied by multidisciplinary expertise and careful monitoring of outcomes.

According to institutional protocol, chest X-ray imaging was routinely performed on POD 3 prior to discharge. In selected patients, including those in the ICU or after later-day surgery, imaging was also obtained on POD 2; if findings were satisfactory, repeat imaging on POD 3 was generally omitted. This variability in imaging timepoints should be considered when interpreting peak pleural effusion values.

Another limitation relates to the extrapolation of data beyond the second week of FU. Since most patients were discharged from FU after their initial control visit, typically between weeks two and four, the number of patients with CXRs available beyond week four was limited. For patients who demonstrated no pleural effusion (0.0% PE) at the first control visit, data were extrapolated under the assumption that they would remain free of PE.

An additional limitation is the numerical imbalance between patients with and without clinically significant PE. Although multivariable logistic regression was used to adjust for potential confounders, the unequal group sizes may have affected the precision and stability of the estimated associations. Future studies could consider propensity score-based approaches to further account for baseline differences between groups.

## Figures and Tables

**Table 1:** Baseline characteristics. Categorical variables are presented in frequencies and (percentages). Continuous variables are presented as mean and (±SD) if distributed normally, and presented as median and [IQR] if distributed non-normally. *p*-values represent differences between the <25% PE and >25% PE groups.

	<25% PE (*n* = 615)	≥25% PE (*n* = 112)	*p*-Value
Sex (men)	518 (84.2%)	98 (87.5%)	0.376
Age (years)	65 ± 9	67 ± 8	*0.028*
Height (cm)	176 [170–181]	176 [170–180]	0.873
Weight (kg)	83 [74–93]	86 [73–96]	0.066
BMI (kg/m^2^)	26.6 [24.5–29.4]	27.9 [24.6–30.4]	*0.018*
eGFR	77 [66–88]	74 [65–88]	0.288
Diabetes	115 (18.7%)	17 (15.2%)	0.374
PAD	41 (6.7%)	7 (6.3%)	0.867
Recent MI	177 (28.8%)	37 (33.0%)	0.369
LVEF			0.436
Good	552 (89.9%)	102 (91.1%)	
Moderate	54 (8.8%)	10 (8.9%)	
Poor	9 (1.5%)	0 (0.0%)	
Atrial fibrillation	36 (5.9%)	6 (5.4%)	0.836
CLD	32 (5.2%)	6 (5.4%)	0.946
Use of diuretics *	77 (12.5%)	14 (12.5%)	0.981
Euroscore II	0.99 [0.76–1.66]	1.10 [0.79–1.93]	0.307
Urgency			0.912
Elective	332 (54.0%)	58 (51.8%)	
Urgent	267 (43.4%)	51 (45.5%)	
Emergency	16 (2.6%)	3 (2.7%)	
Single vessel	409 (66.5%)	68 (60.7%)	0.235
Multivessel	206 (33.5%)	44 (39.3%)	0.235
Use of antithrombotic therapy			
ASA	509 (82.8%)	88 (78.6%)	0.287
P2Y12i	31 (5.0%)	5 (4.5%)	0.796
DAPT	202 (32.8%)	45 (40.2%)	0.132
LMWH	49 (8.0%)	9 (8.0%)	0.980
DOAC	32 (5.2%)	5 (4.5%)	0.743
VKA	3 (0.5%)	0 (0.0%)	0.459
Continued antithrombotic therapy			
DAPT	51 (8.3%)	20 (17.9%)	*0.002*
Other continued antithrombotic therapy	11 (1.8%)	1 (0.9%)	0.494

BMI: body mass index; eGFR: estimated glomerular filtration; PAD: peripheral artery disease; MI: myocardial infarction; LVEF: left ventricular ejection fraction, Good > 50%, Moderate 50–35% and Poor < 35%; CLD: chronic lung disease; ASA: acetylsalicylic acid; DAPT: dual antiplatelet therapy; LMWH: low molecular weight heparin; DOAC: direct-acting oral anticoagulant; VKA: vitamin K antagonist. * Use of diuretics prior to Endo-CAB.

**Table 2:** Perioperative parameters. Categorical variables are presented in frequencies and (percentages). Continuous variables are presented as mean and (±SD) if distributed normally, and presented as median and [IQR] if distributed non-normally. *p*-values represent differences between the <25% PE and >25% PE groups.

	<25% PE (*n* = 615)	≥25% PE (*n* = 112)	*p*-Value
SLVT (min)	83 [58–119]	99 [66–123]	0.188
Drain time (hours)	8.5 [3.9–22.4]	5.9 [3.8–22.1]	0.468
Drain production (mL)	160 [90–314]	150 [90–366]	0.174
Surgical graft revision	4 (0.7%)	0 (0.0%)	0.392
ICU stay(days)	0.5 [0.5–1.0]	0.5 [0.5–1.0]	0.089
Hospital stay (days)	3 [3–4]	4 [3–5]	*<0.001*
Days until first control	21 [19–26]	20 [15–22]	0.088
TD furosemide (mg)	560 [560–840]	560 [560–940]	0.973
Transfusion	22 (3.6%)	14 (12.5%)	*<0.001*
Type of transfusion	(*n* = 22)	(*n* = 14)	
Erythrocytes	13	9	*<0.001*
FFP	11	5	*0.076*
Thrombocytes	11	8	*0.001*

SLVT: single lung ventilation; ICU: intensive care unit; TD: total doses; FFP: fresh frozen plasma.

**Table 3:** Outcomes and changes in treatment plans. Categorical variables are presented in frequencies and (percentages). Continuous variables are presented as mean and (±SD) if distributed normally, and presented as median and [IQR] if distributed non-normally. *p*-values represent differences between the <25% PE and >25%PE groups.

	<25% PE (*n* = 615)	≥25% PE (*n* = 112)	*p*-Value
Pneumonia	18 (2.9%)	6 (5.4%)	0.185
VATS Hemothorax	4 (0.7%)	1 (0.9%)	0.775
Percentage of pleura effusion at first control	6.1 [0.0–13.4]	33.0 [28.3–40.8]	*<0.001*
Change in treatment (intervention)	(*n* = 78 (12.7%))	(*n* = 91 (81.3%))	*<0.001*
Continue furosemide	55 (8.9%)	24 (21.4%)	
Increase furosemide	22 (3.6%)	32 (28.6%)	
Thoracocentesis	2 (0.3%)	35 (31.3%)	

VATS: video-assisted thoracoscopic surgery.

**Table 4:** Risk factors for clinically significant pleural effusion. Variables were statistically significant in the univariate logistic regression analysis if *p* < 0.10. These variables and variables found in the literature were used for multivariate logistic regression with backwards selection based on LR. The model with the best overall accuracy is shown.

	Univariate		Multivariate	
	OR (95% CI)	*p*-Value	OR (95% CI)	*p*-Value
Sex (men)	1.31 [0.72–2.39]	0.377		
Age (years)	1.03 [1.00–1.05]	*0.028*	1.03 [1.00–1.05]	*0.021*
BMI (kg/m^2^)	1.05 [0.99–1.10]	*0.091*	1.06 [1.01–1.12]	*0.027*
Impaired LVEF				
Moderate	1.00 [0.494–2.03]	0.995		
Poor	0.00 [0.00–0.00]	0.999		
Atrial fibrillation	0.91 [0.37–2.21]	0.836		
CLD	1.03 [0.42–2.53]	0.946		
eGFR	0.99 [0.98–1.00]	0.191		
Use of diuretics *	0.99 [0.54–1.83]	0.981		
Euroscore II	1.05 [0.88–1.25]	0.579		
Urgency				
Urgent	1.09 [0.73–1.65]	0.669		
Emergency	1.07 [0.30–3.80]	0.913		
Hospital stay	1.07 [0.97–1.18]	0.185		
Operation time	1.00 [0.99–1.01]	0.231		
Single vessel	0.78 [0.51–1.18]	0.236		
Multivessel	1.29 [0.85–1.95]	0.236		
Continued antithrombotic therapy				
DAPT	2.40 [1.37–4.22]	*0.002*	2.22 [1.19–3.91]	*0.012*
Other anticoagulants	0.50 [0.06–3.87]	0.502		
Single lung ventilation (min)	1.00 [0.99–1.01]	0.196		
Drain time (hours)	0.99 [0.98–1.00]	0.300		
Blood transfusion	3.85 [1.91–7.78]	*<0.001*	3.42 [1.65–7.08]	*0.001*

CLD: chronic lung disease; BMI: body mass index; LVEF: left ventricle ejection fraction; eGFR: estimated glomerular filtration rate; DAPT: dual antiplatelet therapy. * Use of diuretics prior to Endo-CAB.

## Data Availability

Data are available upon reasonable request from the corresponding author.

## Abbreviations

The following abbreviations are used in this manuscript:

Endo-CAB	Endoscopic-Assisted Coronary Bypass Surgery
PE	Pleural Effusion
PCI	Percutaneous Coronary Intervention
ERAS	Enhanced Recovery After Surgery
POD	Postoperative Day
CXR	Chest X-Ray
SD	Standard Deviation
IQR	Interquartile Range
